# New biochemical approaches for the treatment of glioblastoma

**DOI:** 10.1016/j.jbc.2025.110748

**Published:** 2025-09-20

**Authors:** Laura A. Lindsey-Boltz, Aziz Sancar

**Affiliations:** Department of Biochemistry and Biophysics, University of North Carolina School of Medicine, Chapel Hill, North Carolina, USA

**Keywords:** temozolomide, KL-50, gliocidin, 5-Ethynyl-2'-deoxyuridine, EdU, vorasidenib, chronotherapy, KL001, SHP656, SR9009, SR9011, M47

## Abstract

Glioblastoma is a relatively common form of brain tumor for which at present there is no efficient surgical or pharmaceutical treatment. Even with the standard of care consisting of surgery, radiotherapy, plus temozolomide treatment, the median survival time is around 12 months. Recently, several new chemotherapeutic approaches have been developed that target DNA or the circadian clock for treating glioblastoma. Here, after briefly reviewing the standard of care, temozolomide, we summarize the mechanistic bases of these new approaches and their potential for improving treatment of glioblastoma and other brain tumors.

Glioblastoma accounts for approximately 50% of all malignant central nervous system (CNS) tumors in the United States, and despite aggressive standard-of-care treatment, the mean survival remains just around 12 months (1). Unique challenges of treating brain tumors, such as the critical role of the CNS in maintaining the quality of life and the presence of the blood–brain barrier (BBB), which prevents the entry of many conventional chemotherapeutic agents, make treatment of the most aggressive brain tumors, glioblastomas, especially difficult. As a result—or perhaps in response to these limitations—a number of novel chemotherapeutic drugs have been developed in recent years. In this review, we categorize these emerging therapies into two broad classes: DNA-targeting drugs and circadian clock–targeting drugs. We begin with temozolomide (TMZ), a DNA-alkylating agent that has been in use since 1999 and has become the standard of care, albeit with limited long-term success. We then describe recently developed DNA-targeting therapies that act through a variety of mechanisms, including direct DNA damage and disruption of nucleotide or NAD^+^ metabolism, all ultimately leading to cytotoxicity. Finally, we review circadian clock–targeting drugs, which were initially designed to exploit the biological rhythms of tumor and host cells to enhance treatment efficacy. While the original rationale may not fully explain their efficacy, emerging agents that modulate core clock proteins have shown promise as novel strategies to treat glioblastoma and other CNS tumors. It should be noted, however, that, with one exception, these drugs/methods are in experimental (preclinical) stages even though all appear promising for brain tumor treatment in animal studies.

## DNA-targeting drugs

**Figure 1:**
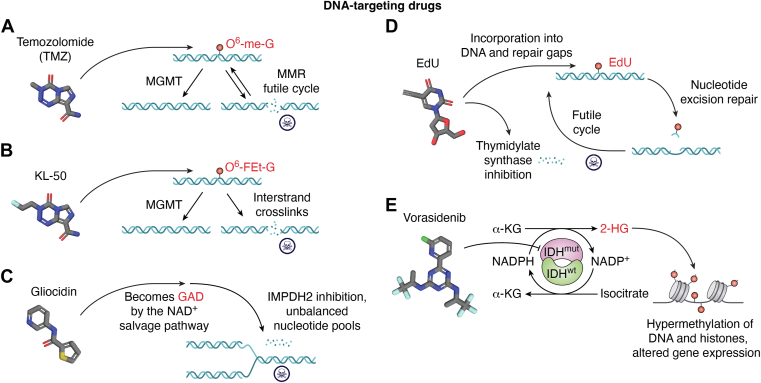
**DNA-targeting drugs.***A* and *B,* both temozolomide (TMZ) and KL-50 induce DNA damage that is repaired by methyl guanine DNA methyltransferase (MGMT). Unrepaired TMZ-induced DNA damage results in cell death because of a futile cycle of mismatch repair (MMR), whereas KL-50-induced damage forms deadly interstrand DNA crosslinks if not repaired within 8 h. *C,* gliocidin enters the NAD^+^ salvage pathway to become the NAD^+^ mimetic, gliocidin-adenine dinucleotide (GAD), which inhibits inosine monophosphate dehydrogenase 2 (IMPDH2), an enzyme essential for guanine synthesis, resulting in unbalanced nucleotide pools and death in replicating tumor cells. *D,* EdU causes unbalanced nucleotide pools by inhibiting thymidylate synthase and is also incorporated into replicating DNA and removed by nucleotide excision repair, creating a futile cycle of synthesis and repair. *E,* mutant isocitrate dehydrogenase (IDH) depletes NADPH, essential for pentose phosphate pathways, to generate D-2-hydoxyglutarate (2-HG) from *α*-ketoglutarate (*α*-KG). The 2-HG oncometabolite inhibits multiple enzymes, the consequences of which include hypermethylation of DNA and histones to promote cell proliferation and disruption of cell differentiation. Vorasidenib inhibits mutant IDH enzymes and significantly decreases the levels of 2-HG in tumor cells. EdU, 5-ethynyl-2'-deoxyuridine.

TMZ in conjunction with surgery and radiation is the standard of care for glioblastoma (1). TMZ is a DNA-alkylating agent that methylates N^7^- and O^6^-positions of guanine (2). These adducted bases can be removed from DNA by alkyl adenine glycosylase and base excision repair, and in addition, O^6^-methylguanine (O^6^-me-G) is directly repaired by the methyl guanine DNA methyltransferase (MGMT) suicide enzyme, which transfers the methyl group from the O^6^-position of guanine to the active site residue of cysteine to repair DNA while inactivating the enzyme. TMZ is rather effective against glioblastoma. However, its effectiveness is due to the cell's failure to remove/eliminate all O^6^-me-G from its genome before replication takes place, leading to O^6^-me-G:T mispairing (Fig. 1*A*). The mismatch repair (MMR) system attempts to correct the mismatch by removing the newly inserted T residue; however, upon refilling the resulting gap in the DNA, the repair/replication polymerases again insert a T across from O^6^-me-G, leading to a “vicious cycle” of T insertion and removal, which eventually leads to cell death, which, in the case of glioblastoma, is the death of the cancer cell (2). However, at least two factors lead to the development of resistance. First, high MGMT expression may be accompanied by low MMR activity in tumor cells, in which case the so-called vicious cycle is not of sufficient strength to kill the cancer cells. Second, in many cases, the initial favorable cancer cell death by MMR-induced apoptosis, after a period, ceases to function because of downregulation and/or inactivating mutations in MMR genes and hence loss of the functional futile mis-insertion/removal cycle that causes cell death. In these cases, even if cells are MGMT^-^ and hence produce high levels of mismatches, in the MMR^−/−^ background, cancer cells become resistant to TMZ, survive, proliferate, and acquire additional mutations.

### DNA crosslinking with O^6^-FEt-G (KL-50)

The synthetic compound imidotetrazine(4a) [also designated KL-50(4a)] was synthesized, and its effect on DNA and potential for glioblastoma were evaluated (3, 4). The small molecule KL-50 reacts with O^6^ of guanine, producing O^6^-(2-fluoroethyl)guanine. The O^6^ adduct can be removed by MGMT; however, within 8 h, the small fraction of O^6^-(2-fluoroethyl)guanine displaces its fluoride and reacts with the complementary cytosine, producing G<>C crosslinks, which are highly toxic to cells (Fig. 1*B*). Since neither MGMT nor MMR can resolve interstrand crosslinks, KL-50 is effective in killing glioblastoma cells regardless of their status in regard to these repair pathways. The drug was found to be effective in MGMT^+^ or MGMT^-^ and MGMT^-^MMR^-^ as tested in glioblastoma cell lines, flank tumors, and, importantly, orthotopic intracranial tumors. Moreover, the therapeutic index of KL50 is reported to be higher than all other crosslinking agents, cisplatin and mitomycin E, which makes it a more general anticancer drug. No clinical trials have been reported yet.

### Gliocidin

A high-throughput screen of ∼2 × 10^5^ small molecules for toxicity in a glioblastoma cell line and a counter screen with low-passage mouse embryonic fibroblasts identified gliocidin [*N*-(pyridin-3-yl)thiophene carboxamide] to be uniquely toxic to glioblastoma cell lines but not the embryonic fibroblasts (5). Analyses of its reaction mechanism revealed that gliocidin is a prodrug that is converted to its tumoricidal metabolite gliocidin–adenine dinucleotide (GAD) by nicotinamide nucleotide adenyltransferase (NMNAT1) of the NAD^+^ salvage pathway (Fig. 1*C*). GAD enters the NAD^+^ pocket of inosine monophosphate dehydrogenase 2 and inhibits NAD^+^-dependent *de novo* guanine nucleotide synthesis, resulting in eventual cell death. When tested on xenografts, it was found to cross the BBB and extend the life of mice with orthotopic glioblastoma. Moreover, it was reported that TMZ induces *nmnat1* expression, and hence the conversion of gliocidin to its active form (GAD), and thus, this virtuous cycle would lead to enhanced efficacy of a gliocidin + TMZ combination. Despite these promising features, another small molecule screen had identified gliocidin as an effective candidate for immunosuppression (6). Thus, at present, toxicological and immunosuppression studies are needed before gliocidin can be tested in clinical trials for glioblastoma treatment in humans.

### 5-Ethynyl-2'-deoxyuridine (EdU)

EdU is commonly used in molecular biology to study DNA replication by detecting its incorporation into DNA (as a thymidine analog) through “click chemistry” with a fluorophore carrying an azide group (7). It readily passes the BBB, and its incorporation into the developing brain (7, 8, 9) and even into the enhancer regions of postmitotic neurons (10, 11) can be detected by click chemistry. In replicating cells, such as intestinal epithelium or tumors in any part of the body, EdU, as opposed to all other thymidine analogs, is preferentially incorporated because it inhibits thymidylate synthase and places either dU or EdU into DNA during replication (12, 13). However, these thymidine analogs in DNA are removed by base excision (uracil) and nucleotide excision (EdU) repair mechanisms, which eventually lead to cell death. Based on these findings, EdU was tested as an antiproliferative agent in glioblastoma cell lines in culture or as subcutaneous xenografts and was found to be effective in both cases (14). It was, however, also found to have toxicity to normal tissues by some unknown mechanism in addition to the partial depletion of thymidine pools (14). A potential answer was provided when it was discovered that DNA containing EdU is recognized by the nucleotide excision repair system as “damage” and is processed as such, being removed in the form of canonical nucleotide excision repair 27-mers (15). This was proposed to lead to a “futile cycle” of removal from DNA, degradation to mononucleotides, and reincorporation, to be removed again, eventually leading to apoptotic cell death by essentially permanent presence of high levels of DNA strand breaks/gaps (Fig. 1*D*). This finding explains, at least in part, the observed toxicity of EdU in cell culture that is not seen with other halogen derivatives of thymidine. Indeed, the potential of EdU for killing proliferating cells was recognized more than a decade ago, and it was shown to induce more strand breaks in glioblastoma cells than TMZ, which is the standard of care for treatment of glioblastoma (14). However, at that time, the cause of EdU-induced strand breaks was unknown, and hence, for the next decade, the idea of EdU as an anticancer agent was not pursued. Following the discovery of excision repair–mediated EdU removal from DNA, the potential of EdU in glioblastoma treatment was re-evaluated using recently developed technological tools including the organotypic brain slice culture (OBSC)–based drug assessment platform (16). It was found that EdU has toxic effects on glioblastoma cell lines, and more importantly, it outperformed TMZ in the treatment of glioblastoma with both high- and low-grade brain cancers in the OBSC platform (17). Importantly, the EdU + TMZ combination exhibited a synergistic effect in both the OBSC platform and against human brain xenografts in mice (in preparation). Further toxicity tests and phase I trials in human volunteers are needed before this combination enters large-scale clinical trials.

### Isocitrate dehydrogenase-targeting

Mutation in one of the two isocitrate dehydrogenases (IDH1 and IDH2) is very common in gliomas (70–80%) (18). Wildtype IDH enzymes catalyze the oxidative decarboxylation of isocitrate to α-ketoglutarate (α-KG), reducing NADP+ to NADPH (Fig. 1*D*). Mutant IDH converts α-ketoglutarate to D-2-hydoxyglutarate, which consumes instead of producing NADPH, resulting in NAD^+^ depletion and disruption of multiple NAD^+^-dependent cellular functions, such as regulation of gene expression, DNA repair, and cell death, and thus promotes tumor growth. However, the mechanism by which the accumulation of D-2-hydoxyglutarate leads to brain tumors is not known (19, 20). Recently, an inhibitor of mutant forms of IDH1 and IDH2 has been synthesized (vorasidenib) and has been approved by the Food and Drug Administration for grade 2 IDH-mutant glioma under the name VORANIGO (21).

## Circadian clock-targeting drugs

For time immemorial, attempts have been made to achieve optimal efficacy of drugs and medical procedures by administering them at certain times of the day. Following the elucidation of the molecular basis of the circadian clock in the past 25 years, these empirical approaches have been, to some extent, replaced by mechanism-based attempts to treat diseases ranging from bronchial asthma to lung cancer, colorectal cancer, and glioblastoma (22, 23). These efforts can be classified under two categories: targeting the circadian clock as a whole or targeting the molecular components of the circadian clock, which, aside from their circadian clock function (generating ca 24-h periodicity in molecular and physiological outputs), also perform functions unique to each of the clock components that are not necessarily rhythmic.

### Chronotherapy

Chronotherapy is the administration of anticancer drugs at the designated time of the day to achieve maximal efficacy. In the past 50 years, chronochemotherapy has been attempted using cisplatin, oxaliplatin, and doxorubicin for ovarian and colorectal cancers (22, 23). Although initially, beneficial effects of these chronotherapy regimens were reported for colorectal cancers, larger studies have not supported the beneficial effects of the chronotherapy regimens (22, 23). Similarly, chronotherapy of glioblastoma with TMZ has been attempted. A retrospective study claimed that TMZ given in the morning conferred longer survival than TMZ given in the evening (24). Based on this finding, a prospective study was conducted, and it was reported to support the conclusion of the retrospective analysis (25). However, a critical analysis of both retrospective and prospective studies did not support the conclusions of the previous analyses (26). Consequently, TMZ chronotherapy is currently not practiced, although some oncologists prefer to administer the drug in the morning hours. In summary, while cancer chronotherapy has yet to demonstrate clear clinical benefit, animal studies are still being conducted with the aim of establishing the optimal form of time-dictated drug administration.

### Clock protein-targeting

The mammalian circadian clock operates through a transcription–translation feedback loop, in which core clock genes are rhythmically expressed, and their protein products, in turn, regulate their own transcription. Essentially, all the core clock proteins, CLOCK, BMAL1, CRY1/2, PER1/2, and the secondary loop proteins, REV-ERBα/β and RORα/β/γ, perform cell growth and proliferative functions outside their circadian rhythm functions (22, 27, 28). Thus, potentially any of the clock proteins could be targeted in a manner that interferes with its physiological role in cellular homeostasis in such a way that it disrupts the proliferation of cancer cells without necessarily disrupting the circadian rhythm of physiology *per se*. With this general view, *in vitro* screening of small-molecule libraries has identified several candidates with potentially anticancer applications.

**Figure 2:**
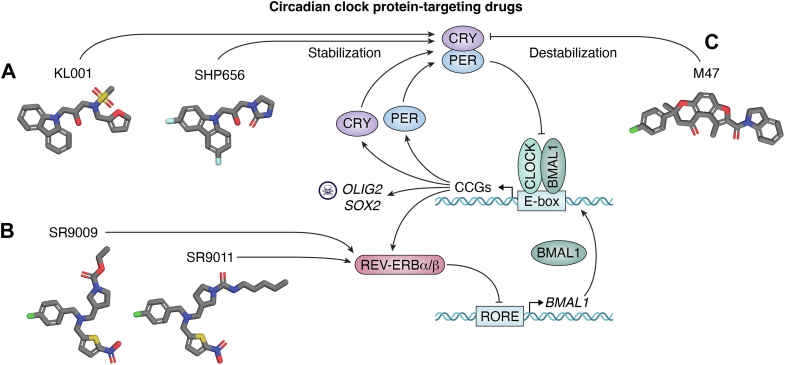
**Circadian clock–targeting drugs.***A,* stabilization of the circadian clock repressor arm (CRY) by KL001 or SHP656 inhibits expression of many of the genes controlled by the CLOCK-BMAL1 transcriptional activator (clock-controlled genes [CCGs]), eventually inhibiting synthesis of proteins required for cell growth, particularly those in glioma stem cells (GSCs). *B,* SR9009 and SR9011 bind to REV-ERBα/β and interfere with its transcription-activating function, resulting in downregulation of cell proliferation. However, it has been reported that these synthetic molecules inhibit tumor cell proliferation independent of REV-ERBα/β as well (36). *C,* M47 inhibits the negative arm of the clock by binding to CRY1, and this disruption of the normal physiological function of the clock in p53 null mutant cells causes apoptosis and stops tumor growth.

KL001, as well as its first-order derivative, SHP656, binds to the “FAD pockets” of CRY1/2 and stabilizes them against proteasomal degradation (Fig. 2*A*). The increased amounts of CRY1/2 bind to BMAL1 and inhibit its transcriptional activity, which subsequently causes a decrease in the expression of clock-controlled genes, including the “stemness” genes (*OLIG2* and *SOX2*), and thus inhibits proliferation of glioma stem cells (GSCs), leading to their cell cycle arrest and eventually apoptosis (29). Similarly, it was reported that SR9011 and SR9009, small-molecule agonists of REV-ERBα/β, which negatively regulate BMAL1, also led to the downregulation of the *OLIG2* and *SOX2* GSC genes and hence reduced expression of their targets (Fig. 2*B*) (29). Moreover, it was reported that when used together, KL001 (CRY1/2 stabilizer) and SR9011 (REV-ERBα/β agonist) exerted a synergistic effect of inhibiting GSC growth as measured by cell-cycle progression arrest and induction of apoptosis (29). Since these small molecules did not affect the circadian clock in general, it was suggested that “the core clock regulators are required for GSC growth, likely by regulating novel biological processes rather than cell-specific modulation of the circadian rhythm” (29).

In an alternative approach, it was also found that inhibiting the negative arm of the clock by destabilizing CRYs may aid in killing cancer cells. The small molecule M47 binds in the “FAD pocket” of CRY1 and selectively enhances its degradation by increasing its ubiquitination (Fig. 2*B*) (27). Use of this drug was found to enhance oxaliplatin-induced apoptosis in Ras-transformed fibroblasts and increase the median lifespan of p53^−/−^ mice by ∼25% (27).

In addition to these synthetic molecules, glucocorticoids have also been used to inhibit glioblastoma growth by enhancing the circadian clock (28, 30, 31, 32). However, recent detailed studies with orthotopic glioblastoma xenografts determined that endogenous corticosteroids exhibit a strong circadian expression pattern (28). However, when the effect of dexamethasone administration to mice with orthotopic xenografts was analyzed, it was found that when the drug was given during early evening (resting phase), when the endogenous glucocorticoid is low, increasing the drug concentration exacerbates glioblastoma growth rates (28, 30). In contrast, when it is administered during the early morning during the peak level of endogenous glucocorticoid, the hormone has only a minor effect on tumor growth. Taking these findings into account, corticosteroids are not recommended for glioblastoma treatment at present.

## Conclusions

Glioblastoma and pancreatic cancer are the two cancers with the lowest survival rates (both having a 5-year survival rate of ∼5%), and these rates have essentially remained constant for the last 25 years (33, 34). In the case of pancreatic cancer, a major factor for low survival is late diagnosis because of the location of the pancreas and because of the vague nature of early symptoms before the cancer has substantially spread (34). In contrast, with glioblastoma, diagnostic symptoms appear relatively early, and so is diagnosis (33). However, the unique nature of the brain and the presence of the BBB, which is either impermeable or poorly permeable to most chemotherapeutic drugs, presents an equally difficult challenge. For these reasons, a number of groups have developed a variety of chemicals that pass the BBB and attack glioblastoma cells. Of these chemicals, those that specifically target the DNA of proliferating tumor cells, such as EdU, are expected to have advantages over other chemotherapeutics that also nonspecifically attack normal brain cells. Beyond the agents covered in this review, several ongoing clinical trials are evaluating additional compounds, such as ascorbate (vitamin C) (35), either alone or in combination with TMZ, with the aim of expanding therapeutic options for this devastating disease.

## Conflict of interest

A.S. has an international patent (WO2024020491) for “Methods of treating cancer of the central nervous system comprising 5-ethynyl-2′-deoxyuridine.” L.A.L.-B. declares no conflicts of interest with the contents of this article.
